# Effect of multi-armed triphenylamine-based hole transporting materials for high performance perovskite solar cells†
†Electronic supplementary information (ESI) available: Experimental details including synthesis, experimental procedure and supporting data. See DOI: 10.1039/c6sc00876c


**DOI:** 10.1039/c6sc00876c

**Published:** 2016-05-17

**Authors:** Sungmin Park, Jin Hyuck Heo, Jae Hoon Yun, Tae Sub Jung, Kyungwon Kwak, Min Jae Ko, Cheol Hong Cheon, Jin Young Kim, Sang Hyuk Im, Hae Jung Son

**Affiliations:** a Photoelectronic Hybrid Research Center , Korea Institute of Science and Technology , Seoul 02792 , Republic of Korea . Email: hjson@kist.re.kr; b Functional Crystallization Center (FCC) , Department of Chemical Engineering , Kyung Hee University , Yongin-si 17104 , Gyeonggi-do , Republic of Korea . Email: imrom@khu.ac.kr; c Department of Chemistry , Chung-Ang University , Seoul 06974 , Republic of Korea; d Department of Chemistry , Korea University , Seoul 02792 , Republic of Korea; e Department of Materials Science and Engineering , Seoul National University , Seoul 02792 , Republic of Korea; f Department of Nanomaterials Science and Engineering , University of Science and Technology (UST) , Daejeon 34113 , Republic of Korea; g KU-KIST Graduate School of Converging Science and Technology , Korea University , Seoul 02841 , Republic of Korea

## Abstract

A series of hole-transporting materials (HTMs) based on [2,2]paracyclophane and triphenyl-amine (TPA) was synthesized. We studied the effect of the chemical structure of the HTM on the photovoltaic performance of perovskite solar cells by varying the number of TPA charge transporting components in the HTM. Tetra-TPA, in which four TPAs are incorporated into the [2,2]paracyclophane core, exhibited better hole transport properties than di-TPA and tri-TPA, which contain two and three TPAs, respectively. In particular, incorporation of the TPA group with a multi-armed structure effectively enhanced the conductivity of the HTM layer in the out-of-plane direction in the solar cell device. Due to the improved charge transport and appropriate molecular energy levels of tetra-TPA, the perovskite solar cell based on the tetra-TPA HTM achieved higher *J*_sc_ and FF values than the devices based on di-TPA and tri-TPA HTMs, with a high solar cell efficiency (17.9%).

## Abstract 2

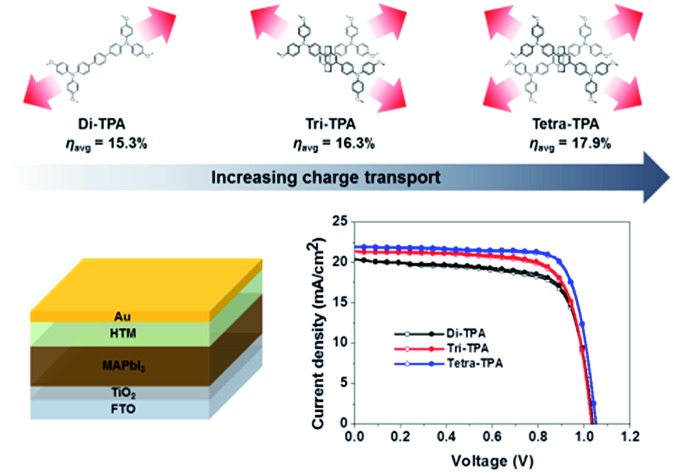
The performance of planar perovskite solar cells was enhanced by using hole transporting materials containing triphenylamine groups with a multi-armed structure.

## 

Organometallic halide perovskite solar cells have emerged as the most promising low-cost photovoltaic technology. The perovskites used in solar cells have the chemical formula ABX_3_, where A, B, and X are ¼CH_3_NH_3_ (or NHCHNH_3_), ¼Pb, and ¼halide (Br, Cl, or I), respectively. This formula has several advantageous properties for the enhancement of photovoltaic effects such as high light absorption, excellent charge carrier diffusion lengths, and a small exciton binding energy.^1^–^5^ Consequently, power conversion efficiencies (PCEs) above 20% have been achieved^6^ that are comparable to those of crystalline Si solar cells.^6^,^7^ Perovskite solar cell devices are typically composed of multiple inter-stacked materials, namely transparent electrodes, electron transporting materials, perovskite photoactive materials, hole-transporting materials, and metal electrodes. Hole-transporting materials (HTMs) are important for achieving high solar cell efficiencies; their roles are to transport holes transferred from the perovskite active layer to the metal electrode and to reduce electron–hole recombination by blocking electron transfer.^8^,^9^ One commonly used HTM is spiro-OMeTAD (2,2′,7,7′-tetrakis[*N*,*N*-di-*p*-methoxyphenylamine]-9,9′-spirobifluorene), which performs well irrespective of the perovskite solar cell device architecture.^7^,^10^–^12^ However, the multi-step synthesis necessary for the preparation of spiro-OMeTAD limits its practical applications in perovskite solar cells.^13^,^14^ Various molecular and polymeric HTMs have been used in perovskite solar cell devices;^2^,^14^–^18^ however, HTM design is hampered by our poor understanding of the relationship between the chemical structures of HTMs and their charge transport properties. It is therefore vital to develop new HTMs and study the relationship between their molecular structures and the photovoltaic properties of solar cell devices based on them. Herein, we present the syntheses of a series of HTMs that incorporate various numbers of a particular transport component, the triphenyl amine group (TPA), and our assessment of the effects of varying the HTM molecular structure on their electrical properties and performances in perovskite solar cell devices.

Tetra-TPA has four TPAs that are incorporated into a [2,2]paracyclophane core, as shown in Fig. 1. [2,2]Paracyclophane has a simple structure that gives HTMs advantageous structural features, such as a cylindrical and rigid structure,^19^,^20^ which promote dense packing. Tri-TPA has one less TPA group than tetra-TPA, while di-TPA is a single component of tetra-TPA that is composed of two *N*,*N*,*N*′′,*N*′′-tetrakis(4-methoxyphenyl)-[1,1′:4′,1′′-terphenyl]-4,4′′-diamine units bridged *via* two ethyl groups. The photovoltaic performances of perovskite solar cell devices depend on the chemical structures of the HTM. It was found that the solar cell device employing tetra-TPA exhibits a higher PCE (17.9%) than the di-TPA-based device (15.3%).

**Fig. 1:**
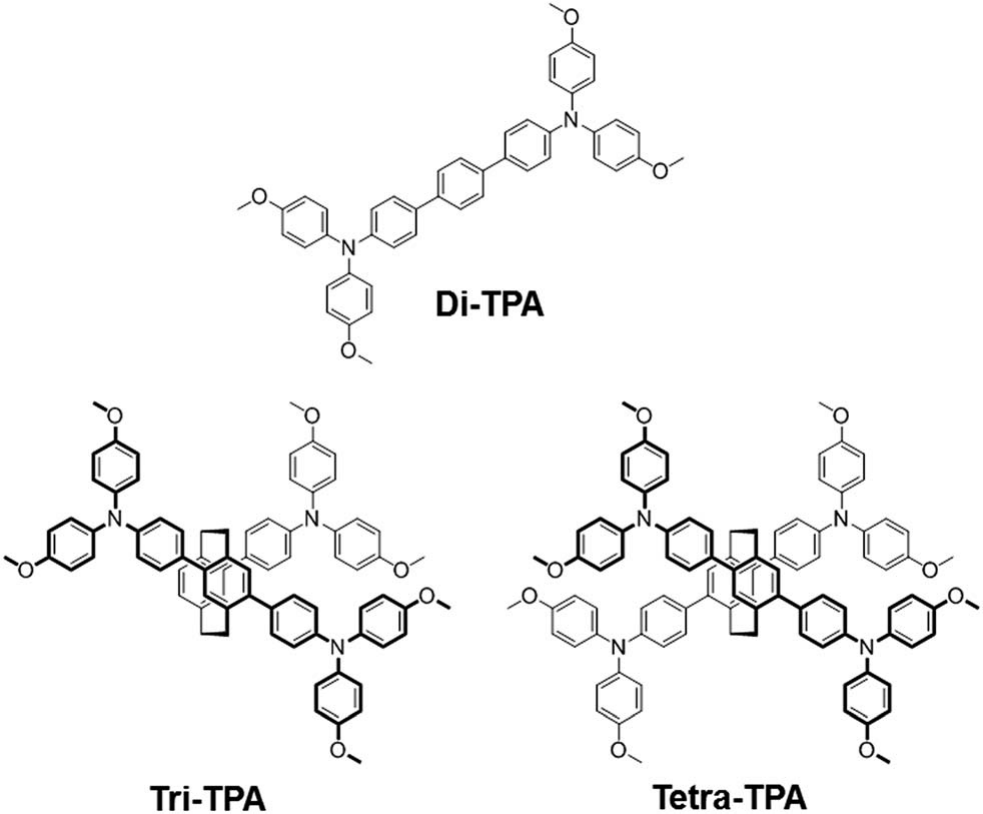
Structures of HTMs containing two, three and four TPAs incorporated into a [2,2]paracyclophane core.

Tetra-TPA and tri-TPA were synthesized by performing the Suzuki cross-coupling reaction of triphenylamine boronic ester with tetra-bromo[2,2]paracyclophane and tri-bromo[2,2]paracyclophane, respectively. The syntheses are described in detail in the ESI.† The UV-vis absorption properties of the HTMs were investigated, as shown in Fig. 2(a) and the characteristic data are summarized in Table 1. The absorption properties of di-TPA and tetra-TPA in solution are very similar, whereas tri-TPA shows a more pronounced first absorption peak at ∼300 nm, with 8–9 nm blue-shifted maximum and onset absorption points compared to those of the other HTMs. The maximum wavelengths of absorption (*λ*_max_) for di-TPA, tri-TPA, and tetra-TPA in toluene are 364 nm, 356 nm, and 365 nm, respectively. The *λ*_max_ absorption values for the HTM films are very similar to the corresponding values for the HTM solutions, although the onset points are red-shifted; interestingly, the red-shifts of di-TPA (13 nm) and tetra-TPA (11 nm) are higher than that of tri-TPA (7 nm), suggesting that the symmetric molecular structures of di-TPA and tetra-TPA are more favorable for intermolecular packing compared to that of asymmetric tri-TPA.^21^ The energy bandgaps (*E*_g_) of the HTMs were calculated from the wavelengths of the intersections of the absorption and emission spectra of the films.^21^ The optical bandgaps of di-TPA, tri-TPA, and tetra-TPA are 2.98 eV, 2.95 eV, and 2.96 eV, respectively. Fig. S10† shows the PL spectra of the three HTMs in solution and in the solid state: the Stokes shifts of tri-TPA and tetra-TPA are ∼20 nm larger than that of di-TPA. In general, the Stokes shift depends on the relaxation to the energy-minimized geometry at the excited state after vertical excitation;^22^,^23^ hence, di-TPA is likely to undergo a smaller geometric relaxation than the other molecules.

**Fig. 2:**
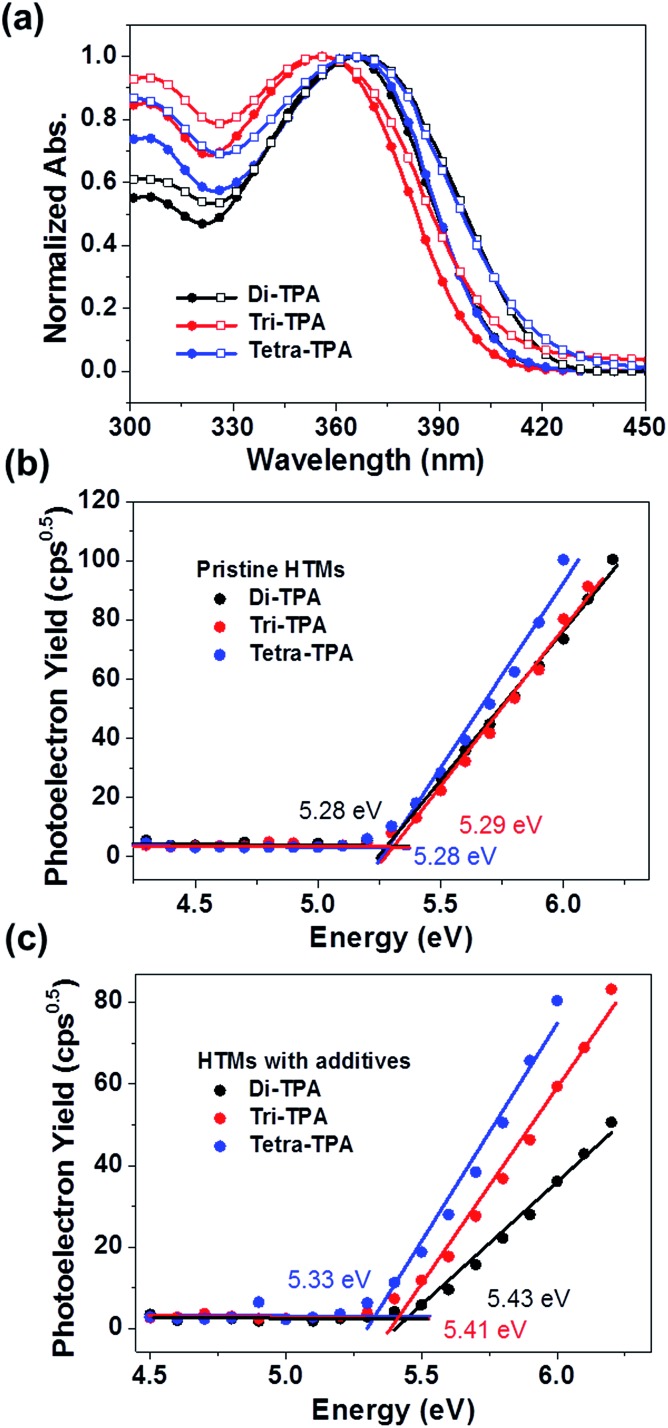
(a) UV/vis spectra of HTMs in toluene ([black circle]) and film (□). (b and c) PESA data of HTMs with and without additives; the lines are linear fits of the data.

**Table 1:** Optical properties of HTMs

HTMs	*λ*_max_ (nm) (solution (film))	*λ*_onset_ (nm) (solution (film))	*λ*_em_ ^*a*^ (nm) (film)	*E*_g_ (eV)
Di-TPA	364 (367)	405 (418)	453	2.98
Tri-TPA	356 (355)	400 (407)	462	2.95
Tetra-TPA	365 (366)	405 (416)	465	2.96

^*a*^Maximum emission excited at *λ*_max_.

The thermal properties of the HTMs were obtained using differential scanning calorimetry (DSC), as shown in Fig. S11.† The glass transition temperatures (*T*_g_) of the HTMs were determined during the second cycle of heating; the values were found to be 81.6 °C, 119.5 °C, and 148.5 °C for di-TPA, tri-TPA, and tetra-TPA, respectively. The *T*_g_ of an organic molecule depends on its rigidity (or flexibility) and can vary due to steric hindrance.^24^ Di-TPA has a relatively low *T*_g_, likely because the *para*-terphenyl core has a higher rotational freedom than the tri-TPA and tetra-TPA cores, *i.e.* [2,2]paracyclophane. Among the three HTMs, tetra-TPA has the highest *T*_g_ because of its dense and rigid structure.

The HOMO energy levels of the HTMs were determined from thin films using photoelectron spectroscopy in air (PESA),^15^,^25^ and their HOMO energy level changes before and after addition of *tert*-butyl pyridine (*t*BP) and lithium bis(trifluoromethylsulfonyl)imide salt (Li-TFSI) additives were compared, as shown in Fig. 2(b and c). Without the additive, all HTMs showed rather similar HOMO values to each other, with –5.28 eV, –5.29 eV, and –5.28 eV for di-TPA, tri-TPA, and tetra-TPA, respectively. However, with the additive, the HOMO energy level of di-TPA was significantly decreased to –5.43 eV, which is a slightly lower value than –5.41 eV of tri-TPA with the additive. Tetra-TPA showed the smallest decrease after adding the additive and thus, the highest HOMO level of –5.33 eV among the HTMs. This is probably because of more effective delocalization of a radical cation over the tetra-TPA molecule, compared with di-TPA and tri-TPA. The reorganization energies (*λ*_h_) of the HTMs, which represent their relaxation after oxidation, were calculated using the density functional theory (DFT) method with the B3LYP functional and 6-311G(d,p) basis set.^26^–^28^ A smaller reorganization energy implies that holes are transferred more efficiently from the perovskite layer to the HTM, as long as the driving force for charge transfer, the HOMO energy offset between the perovskite and HTM layer, is constant. Di-TPA has the lowest calculated reorganization energy (*λ*_h_ = 0.147 eV), followed by tri-TPA (*λ*_h_ = 0.248 eV) and tetra-TPA (*λ*_h_ = 0.595 eV). Therefore, it is expected that the efficiency of hole transfer to the HTM layer decreases in the order di-TPA > tri-TPA > tetra-TPA.

The time-resolved photoluminescence (TR-PL) decays of the HTMs were studied to compare the hole injection into layers of these HTMs from the CH_3_NH_3_PbI_3_ (MAPbI_3_) absorbing layer. PL decay times (*τ*_e_) of the prepared films, where *τ*_e_ is the time required for the PL to fall to 1/e of its initial intensity,^4^ were measured to compare the MAPbI_3_ PL lifetimes of the films, as shown in Fig. 3. The *τ*_e_ value of the pristine MAPbI_3_ is 10.06 ns. When the HTMs without additives were stacked on the perovskite layer, the *τ*_e_ values of MAPbI_3_ were found to be significantly reduced from that of the pristine MAPbI_3_ film and to be dependent on the HTM. In particular, the *τ*_e_ value of tetra-TPA (4.62 ns) is more than twice those of di-TPA (2.08 ns) and tri-TPA (1.57 ns). Hole transfer from the MAPbI_3_ layer to the HTM is more efficient for di-TPA and tri-TPA than for tetra-TPA, which could be because the reorganization energies of di-TPA and tri-TPA are lower than that of tetra-TPA due to the lower energy cost of receiving a hole for these molecules. However, when the additives were included in the HTM, a different trend was found in *τ*_e_: di-TPA and tri-TPA exhibit *τ*_e_ values of 4.54 ns and 4.27 ns, respectively, which are longer than the corresponding values obtained in the absence of the additives. In contrast, the *τ*_e_ value of tetra-TPA is slightly reduced to 3.14 ns after the addition of the additives and is even lower than those of di-TPA and tri-TPA. These results are mainly associated with the HOMO energy levels of the HTMs: the decreases in the HOMO energy levels of di-TPA and tri-TPA are larger compared to that of tetra-TPA after the addition of the additives, which results in smaller energy offsets between the HOMO energy levels of these HTMs and the perovskite layers (valence band edge of MAPbI_3_ = –5.46 eV),^15^ and thus in lower driving forces for charge transfer than that of tetra-TPA. Despite their lower reorganization energies, the smaller driving forces for charge transfer of di-TPA and tri-TPA result in less efficient hole transfer in solar cell devices.

**Fig. 3:**
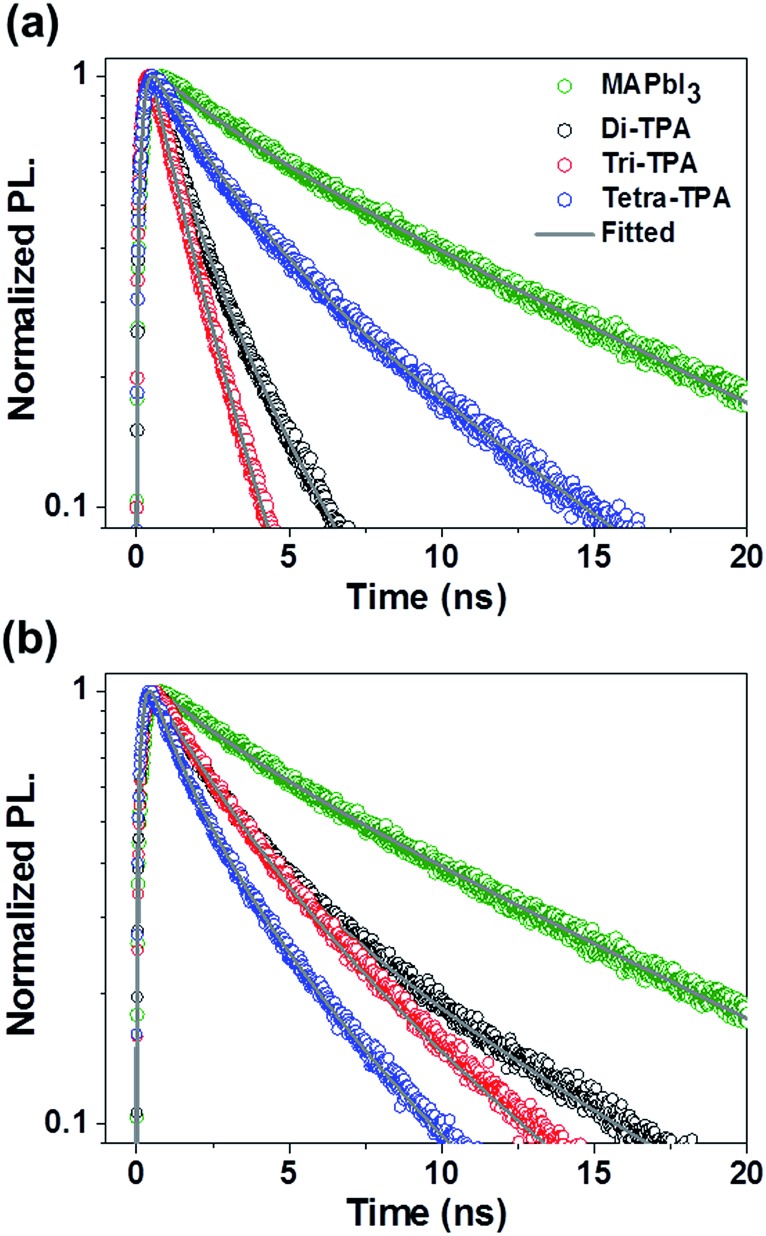
TR-PL decay curves of MAPbI_3_ in the presence of HTMs (a) without and (b) with additives.

The hole mobility values were measured using the space charge limited current (SCLC) method. Tetra-TPA has the largest hole mobility value, 6.32 × 10^–4^ cm^2^ V^–1^ s^–1^, followed by tri-TPA, 5.1 × 10^–4^ cm^2^ V^–1^ s^–1^ and di-TPA, 3.8 × 10^–5^ cm^2^ V^–1^ s^–1^ (Fig. S12†). X-ray diffraction (XRD) data indicated that all the HTMs have similar amorphous properties in films; hence the differences between the mobilities of the HTMs are not related to their film packing structures. Conductive atomic force microscopy (c-AFM) was performed on the HTM films to obtain their current maps. As shown in Fig. 4, there is a higher electric current over the whole tetra-TPA film than for the other films and the di-TPA film exhibits the lowest current. These results indicate that charge transport in the out-of-plane direction decreases in the order tetra-TPA > tri-TPA > di-TPA. Thus, it is concluded that the introduction of the TPA groups to create a multi-armed structure in tetra-TPA is favorable for intermolecular charge transport in the amorphous film.

**Fig. 4:**
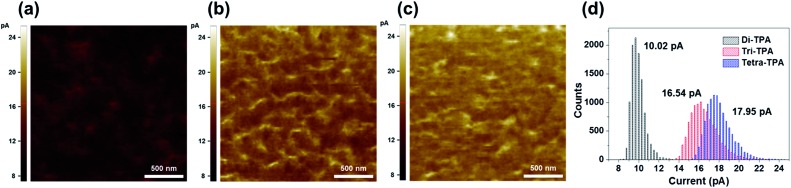
c-AFM current images (size: 2 μm × 2 μm) of (a) di-TPA, (b) tri-TPA, and (c) tetra-TPA on ITO at +1 V. (d) Histograms of currents over the whole area in c-AFM image: letters in (d) refer to mean currents.

Planar MAPbI_3_ hybrid solar cells with a device structure of FTO/TiO_2_/MAPbI_3_/HTM/Au were prepared using the three HTMs. A representative SEM cross-sectional image of these devices is shown in Fig. S13.† Fig. 5 shows the device performances of the planar MAPbI_3_ hybrid solar cells, and their characteristic photovoltaic properties are summarized in Table 2. The device based on tetra-TPA exhibits the best performance, with a PCE (*η*_avg_) of 17.9% averaged over individual measurements under forward and reverse scan conditions, followed by tri-TPA with a PCE (*η*_avg_) of 16.3%. The solar cell device with di-TPA has the lowest efficiency, PCE (*η*_avg_) = 15.3%. Fig. S14(a–c)† shows histograms of the PCE deviations of 40 devices. The average PCE values were 14.5 ± 2.1%, 13.1 ± 2.0%, and 12.0 ± 1.6% for tetra-TPA, tri-TPA, and di-TPA, respectively. The overall efficiency improvement that results from using tetra-TPA rather than di-TPA arises from the increases in the *J*_sc_ and FF values. The external quantum efficiency (EQE) spectra in Fig. 5(d) are consistent with the *J*_sc_ results; the tri-TPA and tetra-TPA devices exhibit higher EQE values in the range of 350–750 nm compared with the di-TPA device. Upon illumination, the MAPbI_3_ layer absorbs light and generates free electrons and holes or loosely bonded electron–hole pairs due to the small exciton binding energy of 30 meV.^4^ Therefore, most electrons and holes are generated in the perovskite active layer and then transported to the TiO_2_ electron conductor and HTM, respectively. The improved *J*_sc_ and FF values of the tetra-TPA-based solar cell are attributed to the more efficient charge transfer from the perovskite layer to the HTM, as expected from the TR-PL results, and the increased charge transport in tetra-TPA. The improved charge transport properties in the device based on tetra-TPA can be attributed, at least in part, to the multi-armed TPA having a greater capacity for efficient charge transport. We also fabricated solar cell devices with spiro-OMeTAD using the same processing conditions employed for the new HTMs. The PCE is an averaged value from the efficiencies obtained from the forward and reverse scans. The best efficiency is 15.65% and the average value obtained from 40 devices is 12.2 ± 1.9%. Fig. S15(a–c)† shows photovoltaic performance, a EQE spectrum, and a PCE histogram of the device. From the results, it is revealed that di-TPA shows similar performance to that of spiro-OMeTAD. Tri-TPA exhibits improved performance due to its enhanced *J*_sc_. The efficiency of the tetra-TPA based solar cell is 2–3% higher due to improvement in the *J*_sc_ and FF values.

**Fig. 5:**
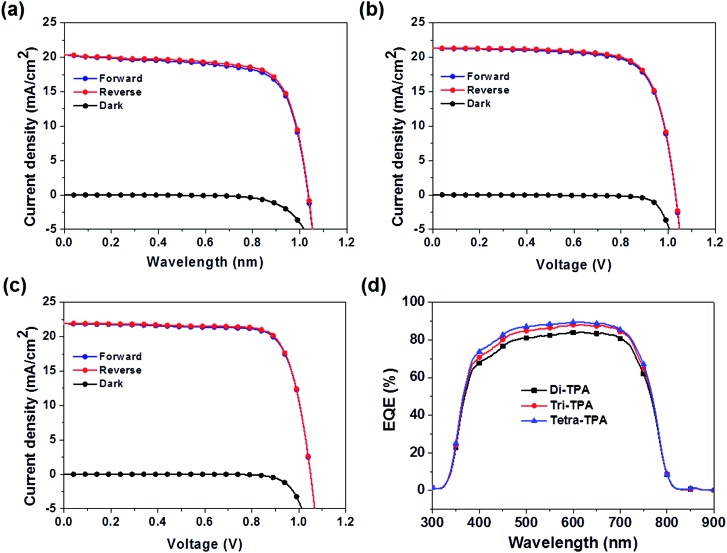
Photovoltaic properties of planar MAPbI_3_ hybrid solar cells with (a) di-TPA, (b) tri-TPA, and (c) tetra-TPA and (d) EQE spectra.

**Table 2:** Summary of photovoltaic properties of planar MAPbI_3_ hybrid solar cells with various HTMs

HTMs	Scan direction	*V*_oc_	*J*_sc_	FF (%)	*η* (%)	*η*_avg_ ^*a*^ (%)
Di-TPA	Forward	1.03	20.4	71.6	15.0	15.3
	Reverse	1.03	20.5	73.3	15.5	
Tri-TPA	Forward	1.03	21.4	73.6	16.2	16.3
	Reverse	1.03	21.4	74.4	16.4	
Tetra-TPA	Forward	1.05	21.8	77.7	17.8	17.9
	Reverse	1.05	22.0	78.0	18.0	

^*a*^PCE values averaged over forward and reverse scans.

## Conclusions

We have developed HTMs with a [2,2]paracyclophane core and investigated the effects of adding TPA units with a multi-armed structure on the photovoltaic properties of perovskite solar cells based on the HTMs. The introduction of the TPA group was found to play an important role in enhancing the charge transport in the amorphous HTM film and thus in improving the perovskite solar cell performance. Due to the efficient charge transfer and transport properties, the perovskite solar cell fabricated with tetra-TPA exhibits higher *J*_sc_ and FF values, and thus a higher solar cell efficiency of 17.9%, compared with the corresponding devices prepared with di-TPA and tri-TPA. The present results provide insights for the development of HTMs and thus for the fabrication of efficient perovskite solar cells.

## Supplementary Material

Supplementary information
