# Supplementary material for: Effect of multi-armed triphenylamine-based hole transporting materials for high performance perovskite solar cells
Source: Chem Sci. 2016 May 17;7(8):5517–22. doi: 10.1039/c6sc00876c (PMC6021790; doi:10.1039/c6sc00876c)
Supplement: Supplementary file 1 [file SC-007-C6SC00876C-s001.pdf]

Electronic Supplementary Material (ESI) for Chemical Science.
This journal is © The Royal Society of Chemistry 2016

Supporting Information

Effect of Multi-armed Triphenylamine-based Hole Transporting Materials for High Performance Perovskite Solar Cells

Sungmin Park,†[ad] Jin Hyuck Heo,†[b] Jae Hoon Yun,†[af] Tae Sub Jung,[c] Kyungwon Kwak,[c] Min Jae Ko,[ag] Cheol Hong Cheon,[d] Jin Young Kim,[e] Sang Hyuk Im*[b] and Hae Jung Son*[af]

[a] Photoelectronic Hybrid Research Center, Korea Institute of Science and Technology, Seoul 02792, Republic of Korea. E-mail: hjson@kist.re.kr

[b] Functional Crystallization Center (FCC), Department of Chemical Engineering, Kyung Hee University, Yongin-si 17104, Gyeonggi-do, Republic of Korea. E-mail: imrom@khu.ac.kr

[c] Department of Chemistry, Chung-Ang University, Seoul 06974, Republic of Korea.

[d] Department of Chemistry, Korea University, Seoul 02792, Republic of Korea.

[e] Department of Materials Science and Engineering, Seoul National University, Seoul 02792, Republic of Korea.

[f] Department of Nanomaterials Science and Engineering, University of Science and Technology (UST), Daejeon 34113, Republic of Korea.

[g] KU-KIST Graduate School of Converging Science and Technology, Korea University, Seoul 02841, Republic of Korea

† These authors contributed equally to this work.

Scheme S1. Synthetic routes of HTMs....5
Fig. S1 ^{1}H-NMR spectrum of Di-TPA in CD_2Cl_2....15
Fig. S2 ^{13}C-NMR spectrum of Di-TPA in CD_2Cl_2....16
Fig. S3 MALDI-TOF mass spectrum of Di-TPA....17
Fig. S4 ^{1}H-NMR spectrum of Tetra-TPA in CD_2Cl_2....18
Fig. S5 ^{13}C-NMR spectrum of Tetra-TPA in CD_2Cl_2....19
Fig. S6 MALDI-TOF mass spectrum of Tetra-TPA....20
Fig. S7 ^{1}H-NMR spectrum of Tri-TPA in CD_2Cl_2....21
Fig. S8 ^{13}C-NMR spectrum of Tri-TPA in CD_2Cl_2....22
Fig. S9 MALDI-TOF mass spectrum of Tri-TPA....23
Fig. S10 PL spectra of HTMs (a) in toluene and (b) films....24
Fig. S11 DSC results of HTMs.....25
Fig. S12 *J vs. V* plots for HTM films (a) with additives and (b) without additives.....26
Fig. S13 A representative SEM cross-sectional image of planar $MAPbI_3$ hybrid solar cells.....27
Fig. S14 Histograms of PCEs of (a) Di-TPA, (b) Tri-TPA, and (c) Tetra-TPA.....28
Fig. S15 (a) Photovoltaic properties of planar $MAPbI_3$ hybrid solar cells with Spiro-OMeTAD and (b) the corresponding EQE spectrum. (c) PCE histogram of 40 solar cell devices.....29

General: Unless stated otherwise, all of chemicals and solvents for the syntheses, the sample preparation, and the process of the device fabrication were purchased from sigma-aldrich and TCI Co., Ltd. and used without further purification. The anhydrous tetrahydrofuran(THF) was prepared by distillation with blue or purple sodium benzophenone ketyl before the reaction. 4-(4,4,5,5-Tetramethyl-1,3,2-dioxaborolan-2-yl)-*N*,*N*-bis(4-methoxyphenyl)aniline **(2)** was prepared by following the synthetic procedures reported in the literatures.[1] All of synthesized products were characterized by measuring the ^{1}H-NMR and ^{13}C-NMR using Bruker Ascend 400 spectrometer in $CDCl_3$ and CD_2Cl_2 at 298K. The chemical shifts of NMR data were reported on the basis of tetramethylsilane (TMS, ^{1}H-NMR), $CDCl_3$ or CD_2Cl_2 (^{13}C-NMR) and expressed as parts per million (ppm).[2] The splitting patterns of ^{1}H-NMR were shown as singlet (s), doublet (d), doublet of doublet (dd), triplet (t), quartet (q) and multiplet (m). To double-check the final products, matrix assisted laser desorption/ionization time-of-flight mass spectrometry (MALDI-TOF MS) was performed with *trans*-2-[3-(4-*tert*-butylphenyl)-2-methyl-2-propenylidene]malononitrile (DCTB) as a matrix. The ultra-violet/visible (UV/vis) absorption spectra of the hole transporting materials (HTMs) were obtained using a PerkinElmer Lambada 35 UV/vis spectrometer at room temperature. The photoluminescence (PL) spectra of HTMs were recorded using a Fluorolog3 spectrofluorometer system of HORIBA scientific. For the optical measurements, the solution samples were prepared by dissolving the HTMs in toluene and the films were prepared by spin coating on a pre-cleaned glass substrate. The differential scanning calorimetry (DSC) data of HTMs were recorded using Q20 system of TA instrument in the temperature range of 30 ^{o}C to 270 ^{o}C at the heating and cooling rate of 10 ^{o}C/min. Photoelectron spectroscopy in air (PESA) data were obtained using Riken Keiki AC-2 surface

analyzer with a 10 nW of UV source. The samples for PESA measurements were spin-coated on the glass substrate as pristine HTMs or doped HTMs. Doped HTMs films were prepared with the same condition for the HTMs layer in solar cell devices.

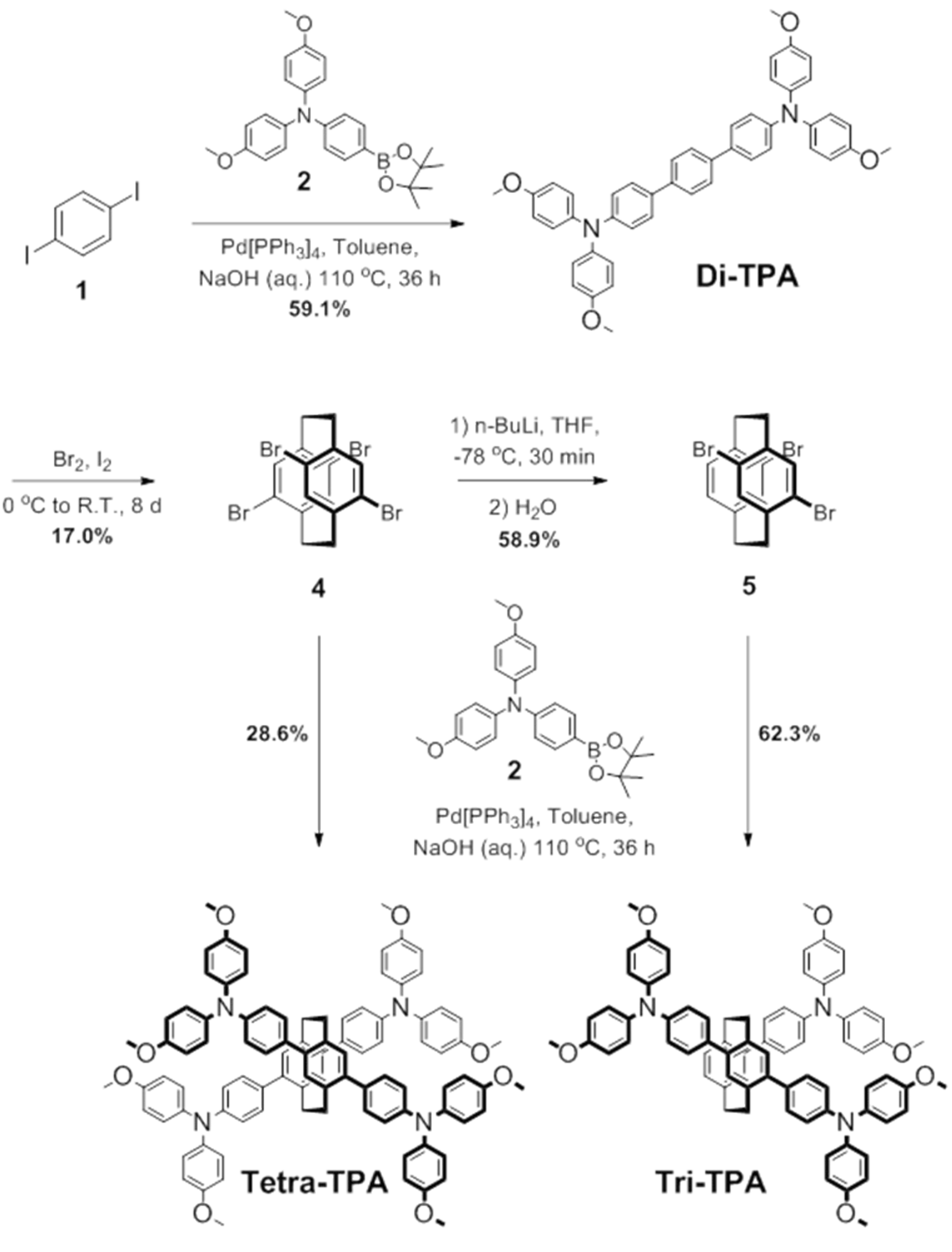

Scheme S1. Synthetic routes of HTMs

Synthesis of HTMs

***N,N,N'',N''*-tetrakis(4-methoxyphenyl)-[1,1′:4′,1″-terphenyl]-4,4″-diamine (Di-TPA)** 1,4-diiodobenzene **(1)** (202.2 mg, 0.61 mmol), 4-(4,4,5,5-tetramethyl-1,3,2-dioxaborolan-2-yl)-*N,N*-bis(4-methoxyphenyl)aniline **(2)** (633.2 mg, 1.47 mmol), and tetrakis(triphenylphosphine)palladium(0) ($Pd(PPh_3)_4$) (70.8 mg, 0.06 mmol) were put into a reaction flask. After purged with argon, deoxygenated toluene (12.3 mL), a sodium hydroxide solution (2 M in deionized water, 3.1 mL), and a drop of aliquat 336 were injected into the flask. The reaction flask was stirred at 110 ^oC for 36 h under argon protection. The crude products were extracted with dichloromethane and brine. The organic layer was dried with anhydrous magnesium sulfate and the solvent was removed using a rotary evaporator. The crude products were purified by silica gel column chromatography using ethyl acetate:hexane=1:9 to obtain the title compound. (247.9 mg, 59.1%) ^1H NMR (400 MHz, CD_2Cl_2) δ 9.60 (s, 4H), 7.46 (d, 4H), 7.08 (d, 8H), 6.96 (d, 4H), 6.85 (d, 8H), 3.79 (s, 12H) ^{13}C NMR (100 MHz, CD_2Cl_2) δ 156.59, 148.71, 141.27, 139.24, 132.77, 127.59, 127.12, 127.00, 120.99, 115.13, 55.89. MALDI-TOF MS m/z (%) calcd. for $C_{46}H_{40}N_2O_4$= 684.299 (100), 685.302 (49.8), 686.306 (12.1), 687.309 (1.1); found 684.159 (100), 685.200 (63.5), 686.161 (20.9), 687.123 (4.6).

4,7,12,15-Tetrabromo[2,2]paracyclophane (4) Iodine (78.7 mg, 0.31 mmol) and bromine (48.15 g, 301 mmol) were added into the reaction flask and cooled down to 0 ˚C using an ice bath. [2,2]Paracyclophane **(3)** (5.23 g, 25.11 mmol) was added into the reaction flask with small portions at 0 ^oC. After addition, the reaction flask was kept with stirring at R.T. for 8

days. And then, the reaction was quenched and neutralized by addition of sodium bisulfide and a sodium hydroxide aqueous solution. The crude product was extracted by chloroform (200 mL) three times and the solution was dried over anhydrous magnesium sulfate. The pure product **(4)** was obtained by column chromatography (eluent: hexane) (2.23 g, 17.0%). ^{1}H NMR (400 MHz, $CDCl_3$) δ 7.17 (s, 4H), 3.18-3.30 (m, 4H), 2.92-3.05 (m, 4H). ^{13}C NMR (100 MHz) δ 140.31, 134.41, 125.28, 32.67.

4,7,12,15-tetrakis-[4-amino-[*N,N*-di-(4-methoxyphenyl)]-phenyl]-[2,2]paracyclophane (Tetra-TPA) 4,7,12,15-Tetrabromo[2,2]paracyclophane **(4)** (806 mg, 1.54 mmol), 4-(4,4,5,5-Tetramethyl-1,3,2-dioxaborolan-2-yl)-*N,N*-bis(4-methoxyphenyl)aniline **(2)** (3.185 g, 7.39 mmol), and tetrakis(triphenylphosphine)palladium (0) ($Pd(PPh_3)_4$) (355.6 mg, 0.31 mmol) were put into a 50 mL flask. After equipped with a condenser, the reaction flask was purged with argon. Deoxygenated toluene (24 mL), a sodium hydroxide solution (2 M in deionized water, 8 mL), and a few drops of aliquat 336 were injected into the reaction flask. The reaction flask was stirred at 110 ^{o}C for 36 h under argon condition. After the reaction, the crude product was diluted with ethyl acetate (200 mL) and washed with water and brine, repeatedly. The organic layer was dried by the adding of anhydrous magnesium sulfate and filteration of the drying agent. The solvent was removed using rotary evaporator under reduced pressure. The pure product **(Tetra-TPA)** was obtained by column chromatography (eluent: ethyl acetate:hexane=1:2) (626 mg, 28.6%) ^{1}H NMR (400 MHz, CD_2Cl_2) δ 7.18 (d, 8H), 7.06 (d, 16H), 6.90 (d, 8H), 6.83 (d, 16H), 6.73 (s, 4H), 3.79 (s, 24H), 3.35-3.60 (m, 4H), 2.65-2.90 (m, 4H). ^{13}C NMR (100MHz) δ 156.42, 147.83, 141.28, 139.58, 137.07, 133.32, 132.47,

130.08, 127.11, 120.65, 115.07, 55.93, 33.78. MALDI-TOF MS m/z (%) calcd. for $C_{96}H_{84}N_4O_8$= 1420.629 (96.3) $[M]^+$, 1421.632 (100), 1422.636 (41.2), 1423.639 (11.3), 1424.642 (4.0); found 1420.409 (97.9) $[M]^+$, 1421.334 (100), 1422.375 (66.2), 1423.416 (30.8), 1424.226 (11.6).

4,7,12-tribromo[2,2]paracyclophane (5) 4,7,12,15-tetrabromo[2,2]paracyclophane **(4)** (300 mg, 0.57 mmol) was dissolved in 10 mL of anhydrous THF and cooled down to -78 ^oC by a dry ice bath. The n-BuLi solution (1.6M, 0.36 mL) was added dropwise into the reaction flask at the same temperature under argon protection. After 30 min, 5 mL of deionized water was added to quench the reaction and the flask was warmed up to room temperature. The crude products were extracted with chloroform and brine. The organic layer was dried with anhydrous magnesium sulfate and the solvent was removed using a rotary evaporator. The crude products were purified by silica gel column chromatography using hexane to obtain the title compound. (150 mg, 58.9%) ^1H NMR (400 MHz, CD_2Cl_2) δ 7.20 (s, 1H), 7.18 (d, 1H), 7.16 (d, 1H), 6.58 (s, 1H), 6.50 (dd, 1H), 3.45-3.20 (m, 3H), 3.18-2.92 (m, 4H), 2.80-2.65 (m, 1H). ^{13}C NMR (100 MHz, CD_2Cl_2) δ 140.88, 140.80, 140.68, 138.54, 138.34, 133.88, 133.29, 131.08, 131.04, 127.18, 125.82, 125.11, 35.25, 33.46, 32.94, 32.39.

4,7,12-tris-[4-amino-[*N,N*-di-(4-methoxyphenyl)]-phenyl]-[2,2]paracyclophane (Tri-TPA) 4,7,12-tribromo[2,2]paracyclophane **(5)** (151.5 mg, 0.34 mmol), 4-(4,4,5,5-Tetramethyl-1,3,2-dioxaborolan-2-yl)-*N*,*N*-bis(4-methoxyphenyl)aniline **(2)** (587.4 mg, 1.36 mmol), and tetrakis(triphenylphosphine)palladium (0) ($Pd(PPh_3)_4$) (78.7 mg, 68.1 μmol) were added into

a 25 mL flask. After equipped with a condenser, the reaction flask was purged with argon. Deoxygenated toluene (6 mL), a sodium hydroxide solution (2 M in deionized water, 2 mL), and aliquat 336 (a few drops) were added into the reaction flask. The reaction flask was stirred at 110 ^{o}C for 36 h under argon protection. After the reaction, the resulting crude product was diluted with ethyl acetate (100 mL) and washed with water and brine, repeatedly. The solution was dried with anhydrous magnesium sulfate and filtered to remove the drying agent. The solvents were removed using a rotary evaporator under reduced pressure. The pure product **(Tri-TPA)** was obtained by column chromatography (eluent: ethyl acetate:hexane=1:3) (236.9 mg, 62.3%) ^{1}H NMR (400 MHz, CD_2Cl_2) δ 7.39 (d, 2H), 7.25 (d, 2H), 7.13 (d, 4H), 7.10-7.03 (m, 10H), 7.00 (d, 2H), 6.94 (d, 2H), 6.88 (d, 4H), 6.86-6.80 (m, 11H), 6.69 (d, 1H), 6.67 (s, 1H), 6.63 (s, 1H), 6.59 (dd, 1H), 3.80 (s, 6H), 3.79 (s, 6H), 3.78 (s, 6H), 3.65-3.50 (m, 1H), 3.50-3.37 (m, 1H), 3.32-3.22 (m, 1H), 3.22-3.10 (m, 1H), 3.01-2.81 (m, 2H), 2.62-2.45 (m, 2H). ^{13}C NMR (100 MHz, CD_2Cl_2) δ 156.55, 156.45, 148.13, 147.90, 141.35, 140.99, 139.68, 139.08, 137.46, 137.34, 136.98, 134.62, 133.78, 133.53, 133.34, 132.49, 130.97, 130.44, 130.12, 130.02, 129.69, 127.23, 127.03, 126.98, 120.87, 120.67, 120.59, 115.18, 55.84, 34.89, 34.57, 34.00, 33.88. MALDI-TOF MS m/z (%) calcd. for $C_{76}H_{67}N_3O_6$= 1117.503 (100), 1118.506 (82.2), 1119.510 (33.3), 1120.513 (8.9), 1121.516 (1.4); found 1117.286 (100), 1118.311 (85.2), 1119.337 (37.2), 1120.261 (12.2), 1121.288 (3.8).

Reorganization energy calculation

The reorganization energy(λ_h) was calculated by the following equation:

$\lambda_h = \left(E^{+}_{0} - E^{+}_{+}\right) + (E^{0}_{+} - E^{0}_{0})$

Here, E^{+}_{0} is the energy of the cation with the optimized structure of the neutral molecule and E^{+}_{+} is the energy of the optimized cation. With these, the structural relaxation energy is calculated when an electron is detached. In the opposite way, E^{0}_{+} is the energy of the neutral molecule with the optimized structure of the cation and E^{0}_{0} is the energy of the optimized neutral molecule. With these, the structural relaxation energy is calculated when an electron is attached to the cation. All of calculations were performed with the Gaussion 09 program package with B3LYP functional and 6-311G(d,p) basis level. First of all, Di-TPA, Tri-TPA and Tetra-TPA were optimized in the neutral and cation states and the energy for each optimized structure is recorded. Then, an electron is removed from the neutral state or added to cation state. The energies of the excited cation (an electron removed from the neutral state) and the excited neutral (an electron is added to the cation) are calculated without further energy optimization to reflect the structure before the energy relaxation.

	E^{+}_{0} (hartree)	E^{+}_{+} (hartree)	E^{0}_{+} (hartree)	E^{0}_{0} (hartree)	λ_h(eV)
Di-TPA	-2187.768	-2187.770	-2187.970	-2187.973	0.147
Tri-TPA	-3552.784	-3552.788	-3552.984	-3552.989	0.248
Tetra-TPA	-4530.851	-4530.681	-4530.505	-4530.697	0.595

Photoluminescence lifetime measurements

Time-resolved PL (TR-PL) results were recorded using an inverted-type scanning confocal microscope (MicroTime-200, PicoQuant) with a 20x objective. For the excitation of the

$MAPbI_3$, a 470 nm pulsed diode laser was used with a pulse width of ~100 ps and a fluence of < 1 μW. The PL was collected using a dichroic mirror (490 DCXR, AHF), a longpass filter (HQ500lp, AHF), a 50 μm pinhole, and a single photon avalanche diode (PDM series, MPD) through a 700 nm long-pass filter. The exponential fitting of PL decays were obtained using the Symphotime v5.3 software. The PL lifetimes of $MAPbI_3$ and with HTMs were compared by τ_e and defined as the time taken after excitation for the decrease to 1/e of the initial population. The samples for TRPL were prepared by the same procedures and conditions of solar cell devices on the glass substrate.

Hole Mobility Measurement

ITO/PEDOT:PSS/HTM layer/Au devices for HTMs were prepared as a hole-only device. The HTM layers contain *t*BP and Li-TFSI additives in concentrations that yield the best solar cells. The hole mobilities of HTMs were calculated from *J-V* curve of the hole-only device in the dark condition by the space-charge-limited current (SCLC) method by using the following equation:

$$J = \frac{9}{8}\varepsilon_0\varepsilon_r\mu_h\frac{V^2}{L^3}$$

In the equation, ε_0 is the vacuum permittivity, ε_r is the dielectric constant of HTMs, μ_h is the hole mobility, V is the voltage drop across the device, and L is the thickness of the HTM layer; the thickness of the HTM layers were averaged of over 5 times measurements using an alpha-step. The thicknesses were 200 nm for Di-TPA and Tri-TPA, and 137 nm for Tetra-TPA. The internal voltage in the device (V) was defined as $V = V_{appl} - V_r - V_{bi}$, where V_{appl} is the

applied voltage to the device, V_r is the voltage drop due to constant resistance and series resistance across the electrodes, V_{bi} is the built-in voltage due to the difference in work functions of the two electrodes.[3,4] The dielectric constant (ε_r) of organic molecules are typically assumed to be 3 and the value was used in this analysis. The current density *vs.* voltage (*J*-*V*) characteristics were obtained at a dark condition using a Keithley model 2400 source measuring unit. For comparsion, hole-only devices of the HTMs without the addtive were also fabricated using the same condition. The thicknesses were 90 nm for Di-TPA and 200 nm for Tri-TPA and Tetra-TPA. The calculated mobilities are 2.1×10^{-5} cm^2/V·s, 5.0×10^{-5} cm^2/V·s, and 6.9×10^{-5} cm^2/V·s for Di-TPA, Tri-TPA, and Tetra-TPA, respectively.

Conductive atomic force microscopy (c-AFM) measurements

The samples for c-AFM were prepared on a pre-cleaned ITO by spin-casting. The thickness of the HTMs films were carefully controlled to obtain same by adjusting the concentration of HTMs. And the ratios of the HTMs and additives (toluene and acetonitrile) were same as those of the HTM layers for the best solar cells. The average thicknesses were obtained by 7 times measurements of the AFM non-contact line profile mode with scratched HTM layer and the values were 90 nm for three HTMs. The c-AFM images for HTMs were obtained using XE-100 AFM of Park systems inc. with contact mode. All images were obtained using a single Pt-Ir coated cantilever (PPP-CONTSCPt, Nanosensors™) by the same measurement conditions. The external current amplifier (DLPCA-200, FEMTO®) was employed and the transimpedance gain was 10^9 V/A.

Syntheis of $CH_3NH_3PbI_3$ ($MAPbI_3$) perovskite solution

To synthesize the MAI, 50 mL hydroiodic acid (HI, 57% in water, 50 mL, Lot:MKBS1107V, Aldrich) and 50 mL methylamine (40% in methanol, 500 mL, Lot:5VT7EQH, Junsei Chemical Co. Ltd.) were reacted in a 250 mL flask at 0 ^{o}C for 2 h with vigorous stirring. The precipitated product was recovered by evaporation of the solvents at 50 ^{o}C for 1 h using a rotary evaporator. For purity of MAI, the resulting product was dissolved in ethanol, recrystallized from diethyl ether, and finally dried at room temperature under reduced pressure oven for 24 h. A 40 wt% of $MAPbI_3$ solution was then prepared by mixing equimolar MAI and PbI_2 (50 g Lot:BCBP521V, Aldrich) (1:1 mole ratio) in *N,N*-dimethylformamide (DMF, 1L, Lot:SHBF6963V, Aldrich) at 60 ^{o}C for 30 min and then, hydriodic acid (HI, 57% in water, 50 mL, Lot:MKBS1107V, Aldrich) was added into the $MAPbI_3$ perovskite solution.

Fabrication of perovskite solar cell devices

To fabricate the $MAPbI_3$ planar hybrid solar cells, a ~50 nm-thick dense TiO_2 electron conductor was deposited on a cleaned F-doped SnO_2 (FTO, Pilkington, TEC8) glass substrate by a spray pyrolysis deposition (SPD) method with a 40 mL of 20 mM of titanium diisopropoxide bis(acetylacetonate) (Aldrich)/iso-propanol solution at 450 ^{o}C. To form the $MAPbI_3$ layer on the bl-TiO_2/FTO substrate, the 0.1 mL of 40 wt% $MAPbI_3$/DMF solution with a HI solution (40 wt% $MAPbI_3$ solution in DMF/HI = 1 mL/0.1 mL) was spread over the entire surface and then spin coated at 3000 rpm for 200 s. The resulting film was dried on a hot plate at 100 ^{o}C for 2 min in air under 36% relative humidity. HTM layers were formed by spin-coating 0.1 mL of the Di-TPA, Tri-TPA, or Tetra-TPA solution in toluene (1 L,

Lot:SHBG0205V, anhydrous, 99.8%, Aldrich : 60 mg HTM in 1 mL toluene) containing 25 μL of a Li-TFSI (170 mg Li-TFSI in 1 mL acetonitrile) and 40 μL of *t*-BP (1 mL 4-*tert*-butyl pyridine in 1 mL acetonitrile) additive solutions at 2000 rpm for 30 s. Finally, a 60 nm-thick Au counter electrode was deposited by thermal evaporation. We used all chemicals as received. The active area was fixed to 0.16 cm^2.

Device characterization

For the characterization of photovoltaic properties, the current density-voltage (*J*-*V*) curves were measured by a solar simulator (Peccell, PEC-L01) with a potentiostat (IVIUM, IviumStat) under illumination of 1 sun (100 mW/cm^2 AM 1.5G) and a calibrated Si-reference cell certified by JIS (Japanese Industrial Standards). The standard condition of scan rate was fixed to 200 ms/10 mV irrespective of the scan direction. The *J*-*V* curves of all devices were measured by masking the active area with a metal mask of 0.096 cm^2. The external quantum efficiency (EQE) was measured by a power source (ABET 150W Xenon lamp, 13014) with a monochromator (DONGWOO OPTORN Co., Ltd., MonoRa-500i) and a potentiostat (IVIUM, IviumStat).

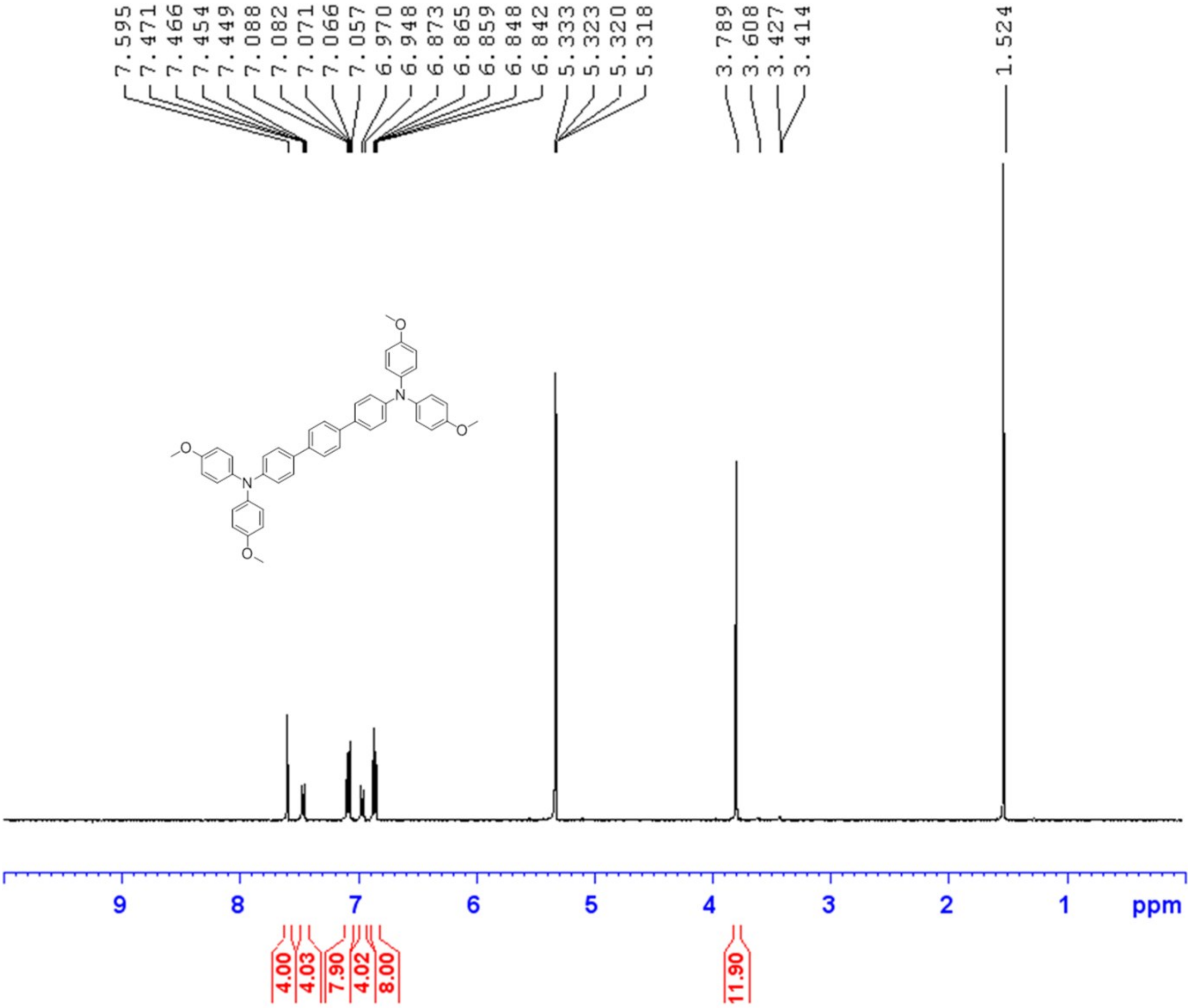

Fig. S1 ^{1}H-NMR spectrum of Di-TPA in CD_2Cl_2

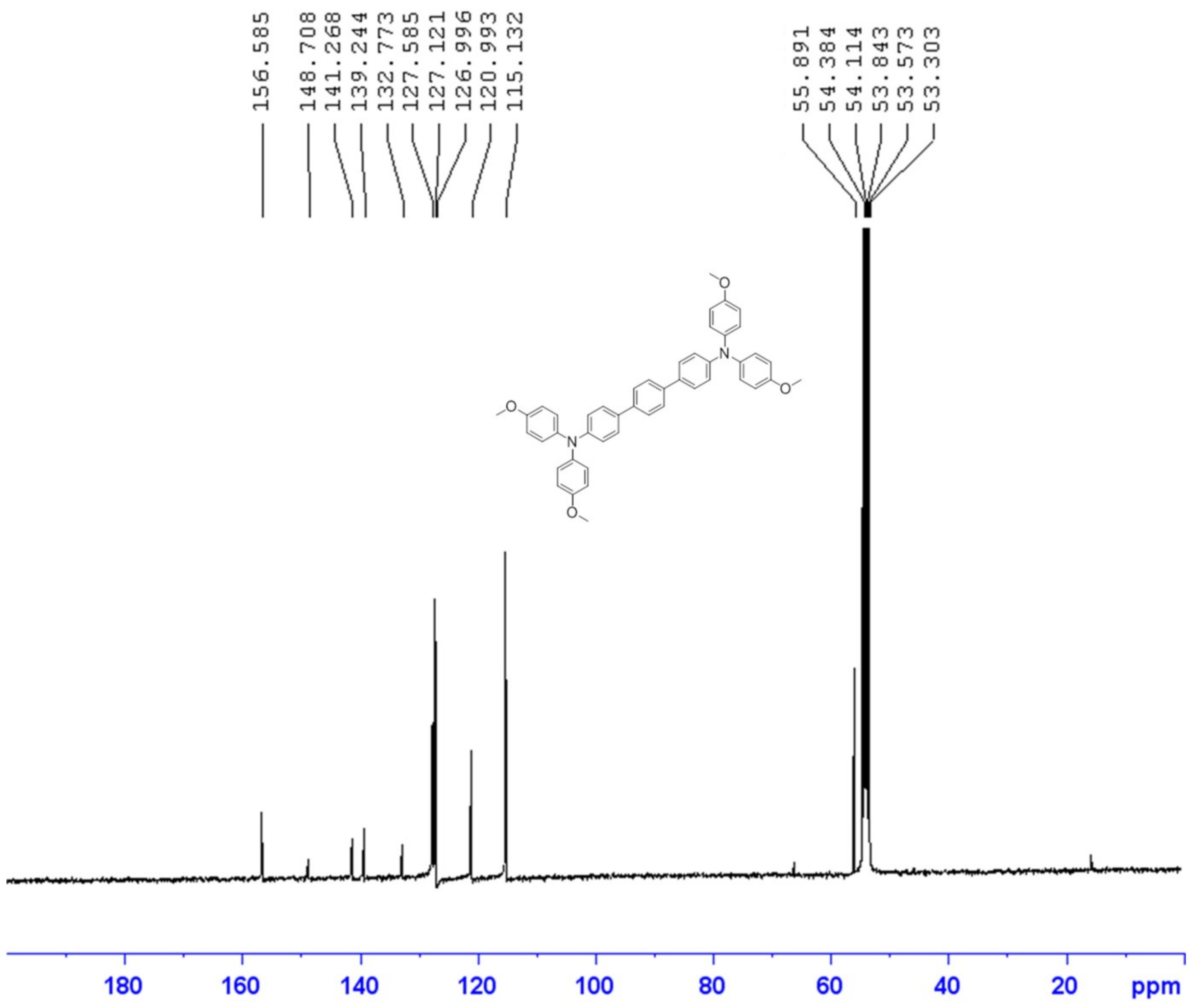

Fig. S2 ^{13}C-NMR spectrum of Di-TPA in CD_2Cl_2

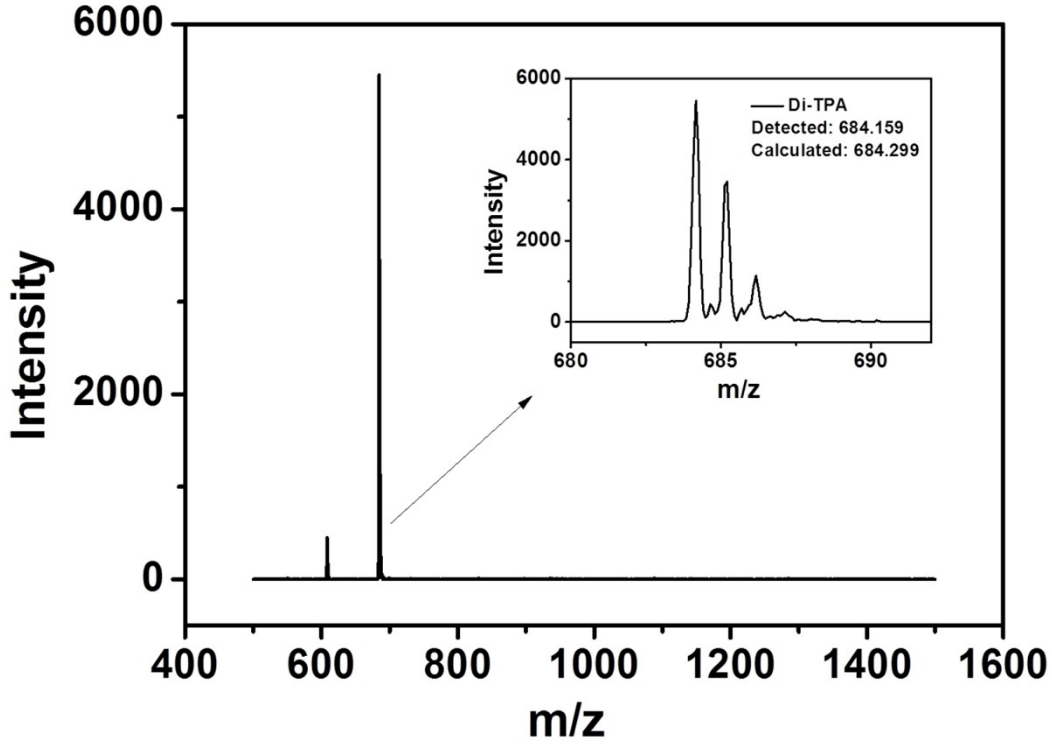

Fig. S3 MALDI-TOF mass spectrum of Di-TPA

: MALDI-TOF MS m/z (%). calcd. for $C_{46}H_{40}N_2O_4$=684.299 (100) $[M]^+$, 685.302 (49.8), 686.306 (12.1), 687.309 (1.1); found 684.159 (100) $[M]^+$, 685.2 (63.5), 686.161 (20.9), 687.123 (4.6).

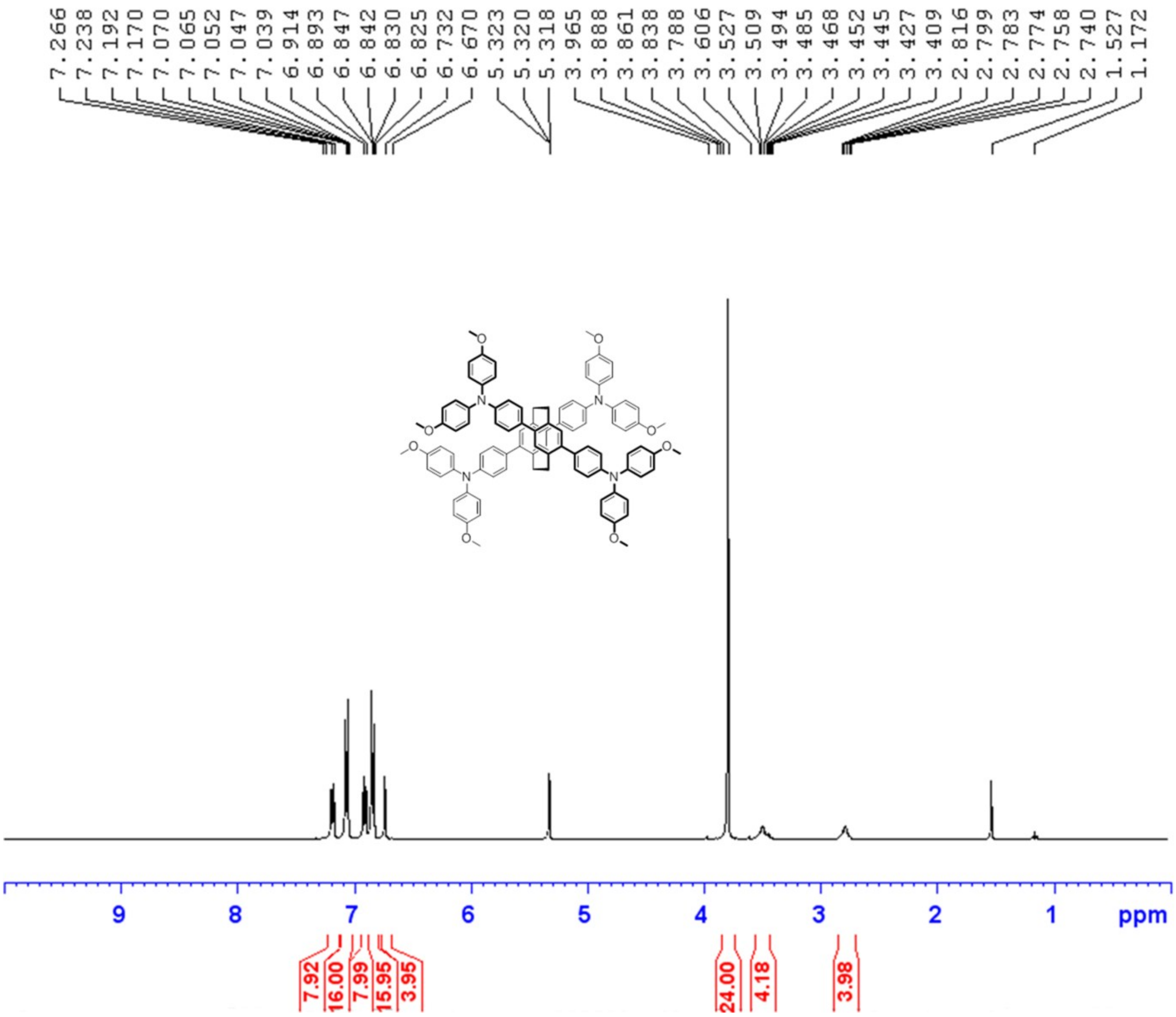

Fig. S4 ^{1}H-NMR spectrum of Tetra-TPA in CD_2Cl_2

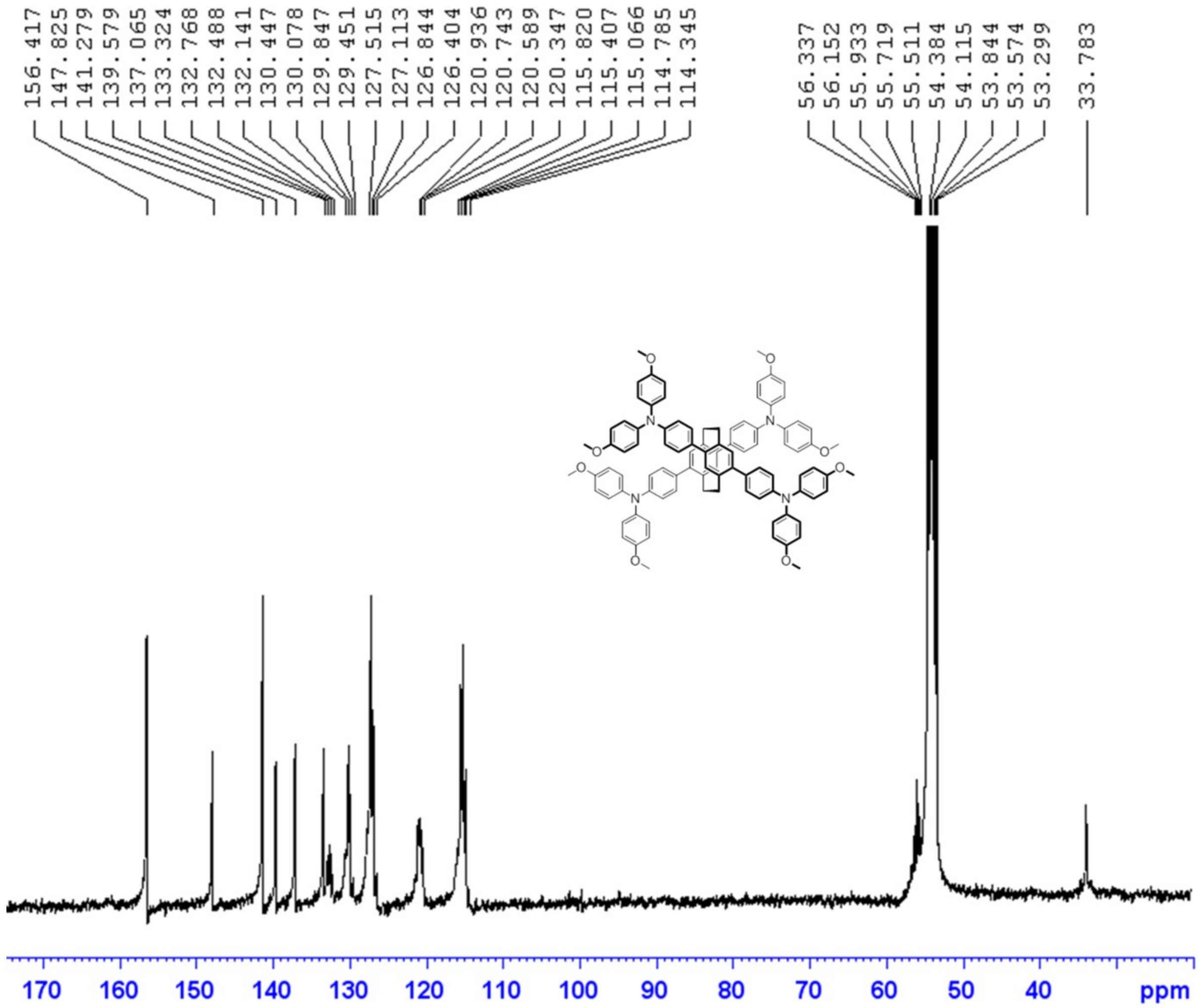

Fig. S5 ^{13}C-NMR spectrum of Tetra-TPA in CD_2Cl_2

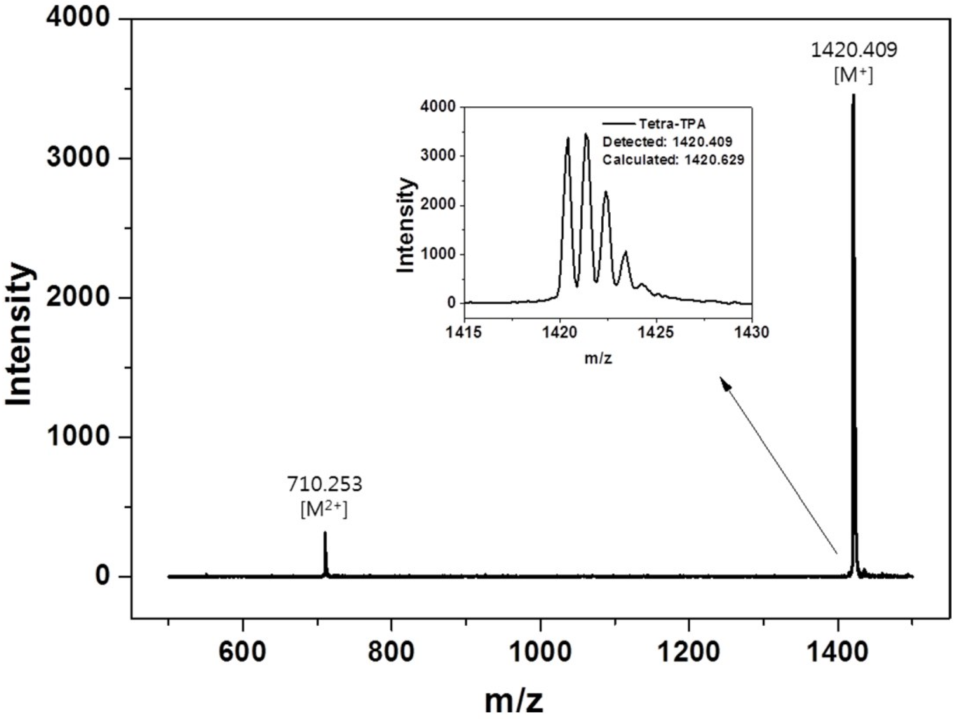

Fig. S6 MALDI-TOF mass spectrum of Tetra-TPA

: MALDI-TOF MS m/z (%). calcd. for $C_{96}H_{84}N_4O_8$=1420.629 (96.3) $[M]^+$, 1421.632 (100), 1422.636 (41.2), 1423.639 (11.3), 1424.642 (4.0); found 1420.409 (97.9) $[M]^+$, 1421.334 (100), 1422.375 (66.2), 1423.416 (30.8), 1424.226 (11.6).

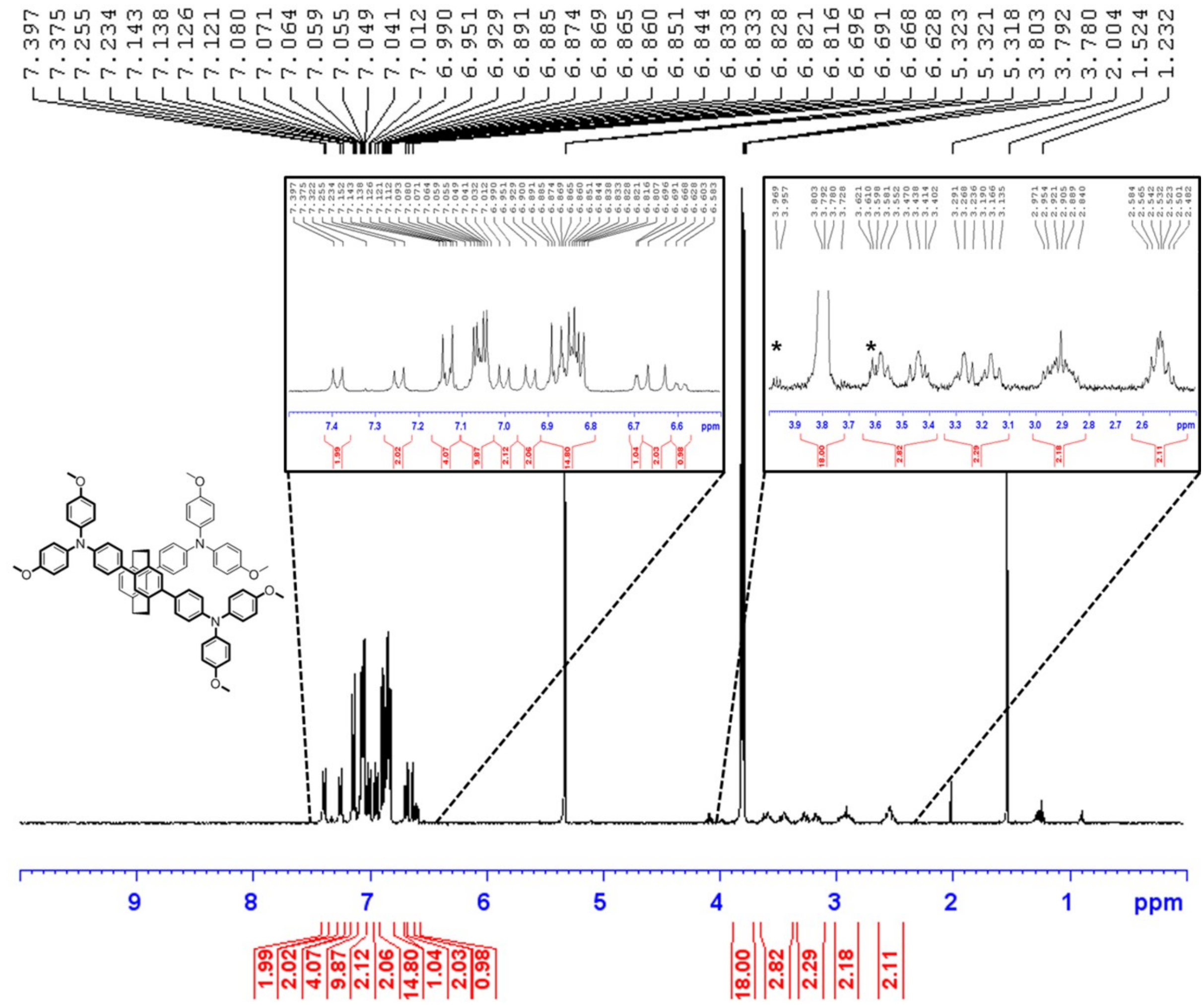

Fig. S7 ^{1}H-NMR spectrum of Tri-TPA in CD_2Cl_2

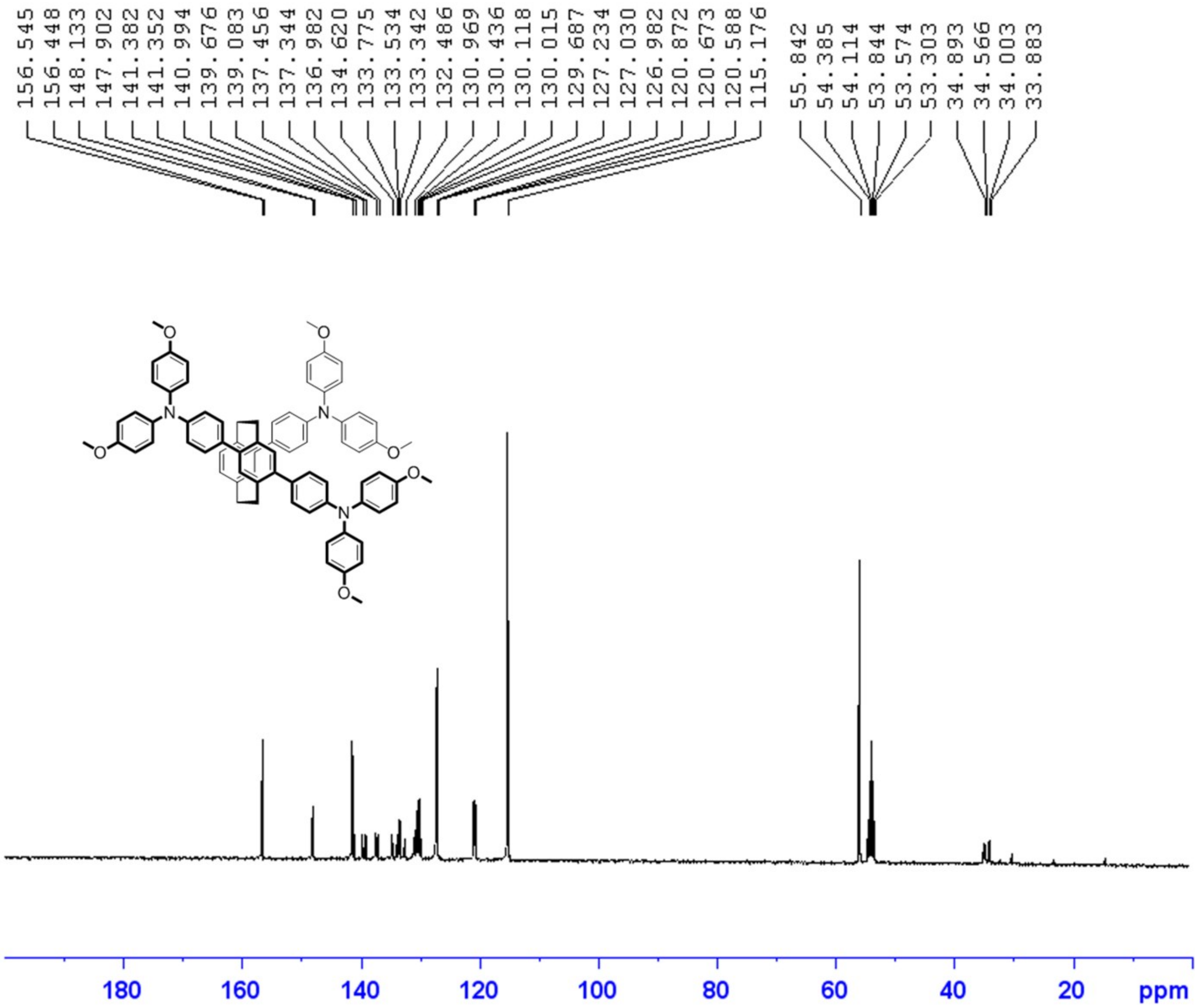

Fig. S8 ^{13}C-NMR spectrum of Tri-TPA in CD_2Cl_2

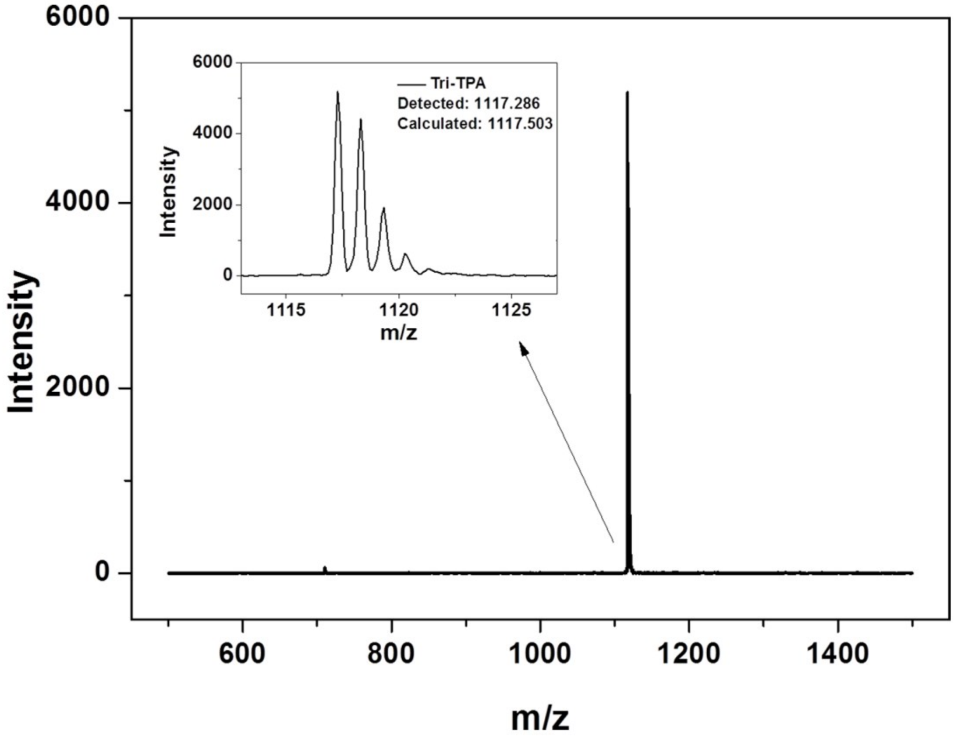

Fig. S9 MALDI-TOF mass spectrum of Tri-TPA

: MALDI-TOF MS m/z (%) calcd. for $C_{76}H_{67}N_3O_6$= 1117.503 (100) $[M]^+$, 1118.506 (82.2), 1119.510 (33.3), 1120.513 (8.9), 1121.516 (1.4); found 1117.286 (100) $[M]^+$, 1118.311 (85.2), 1119.337 (37.2), 1120.261 (12.2), 1121.288 (3.8).

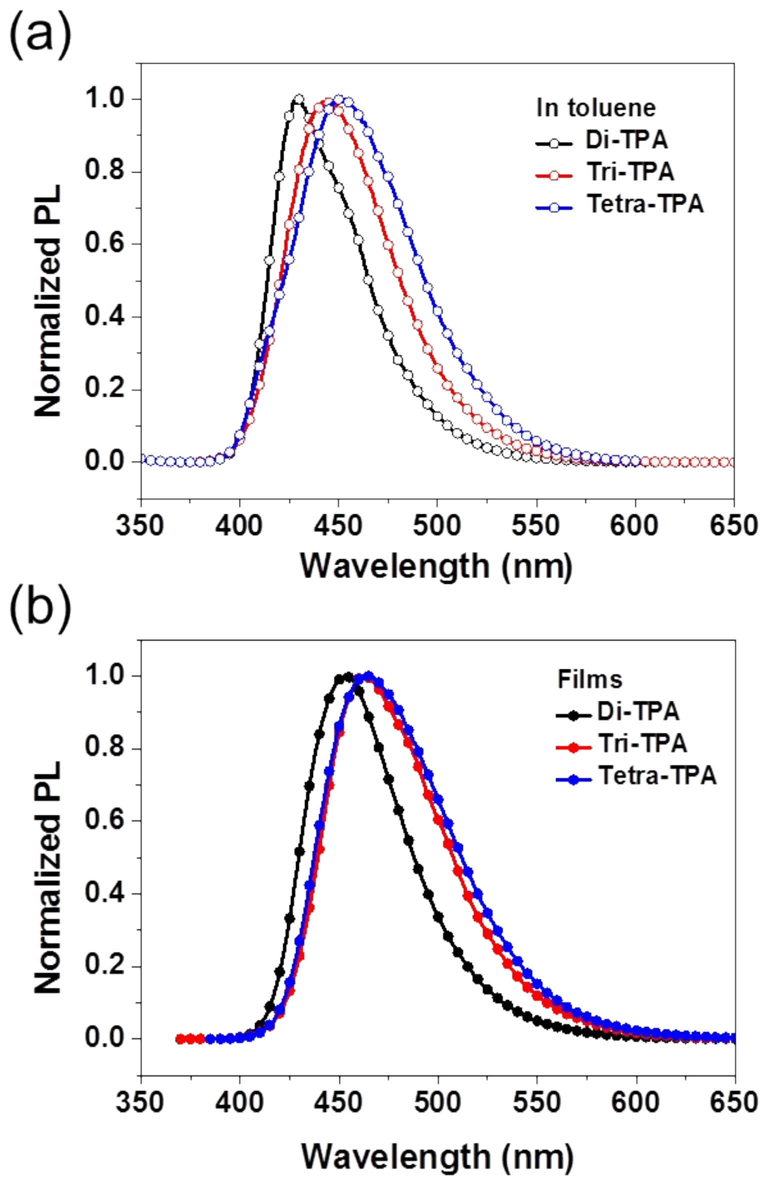

Fig. S10 PL spectra of HTMs (a) in toluene and (b) films

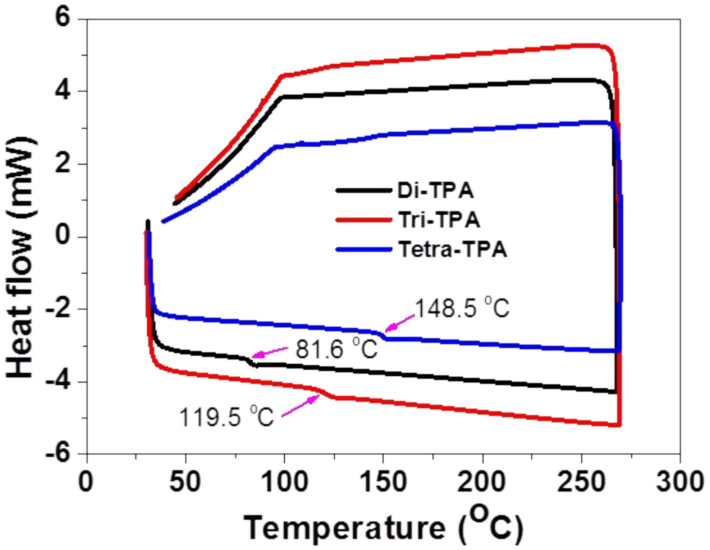

Fig. S11 DSC results of HTMs.

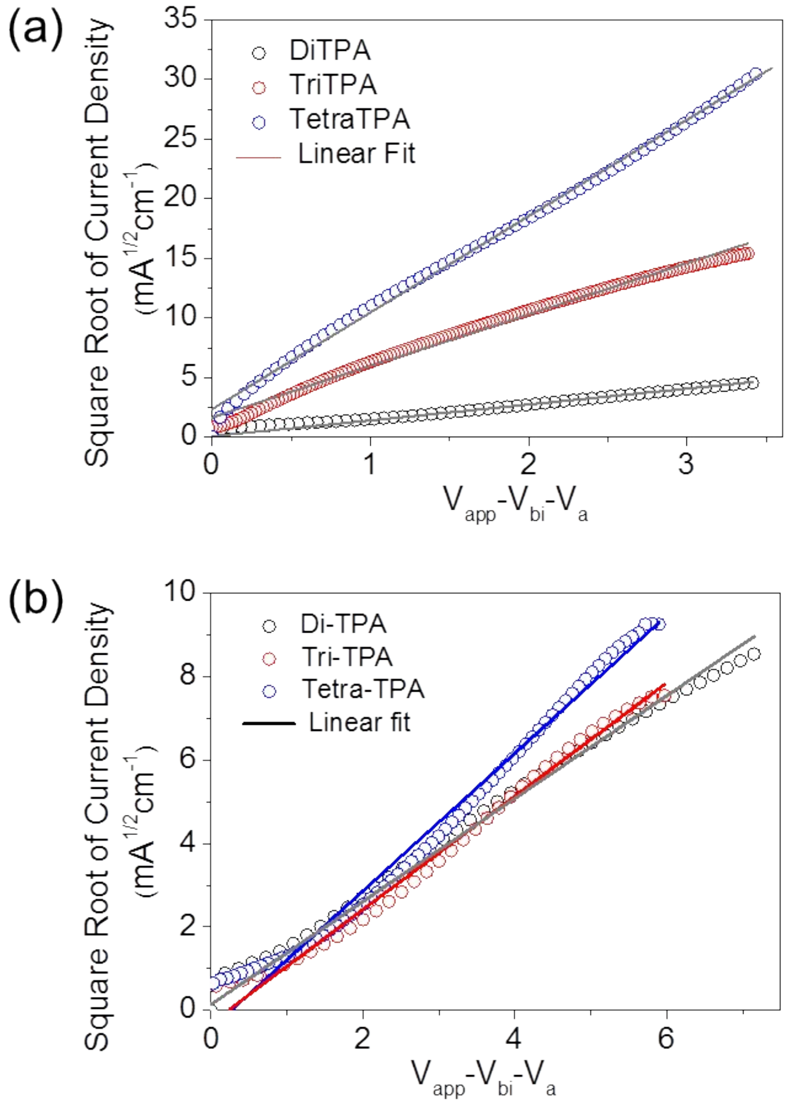

Fig. S12 *J vs. V* plots for HTM films (a) with additives and (b) without additives.

: The solid lines are fits of the data points.

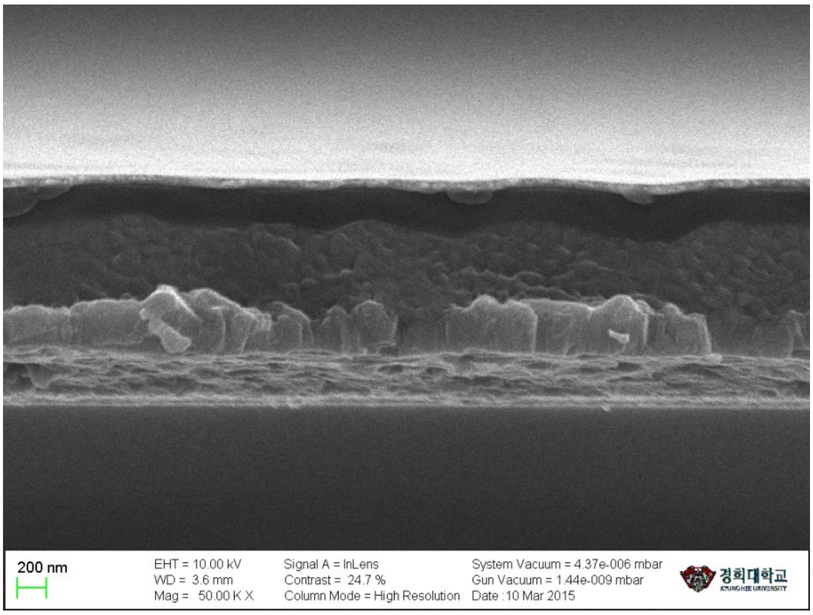

Fig. S13 A representative SEM cross-sectional image of planar $MAPbI_3$ hybrid solar cells.

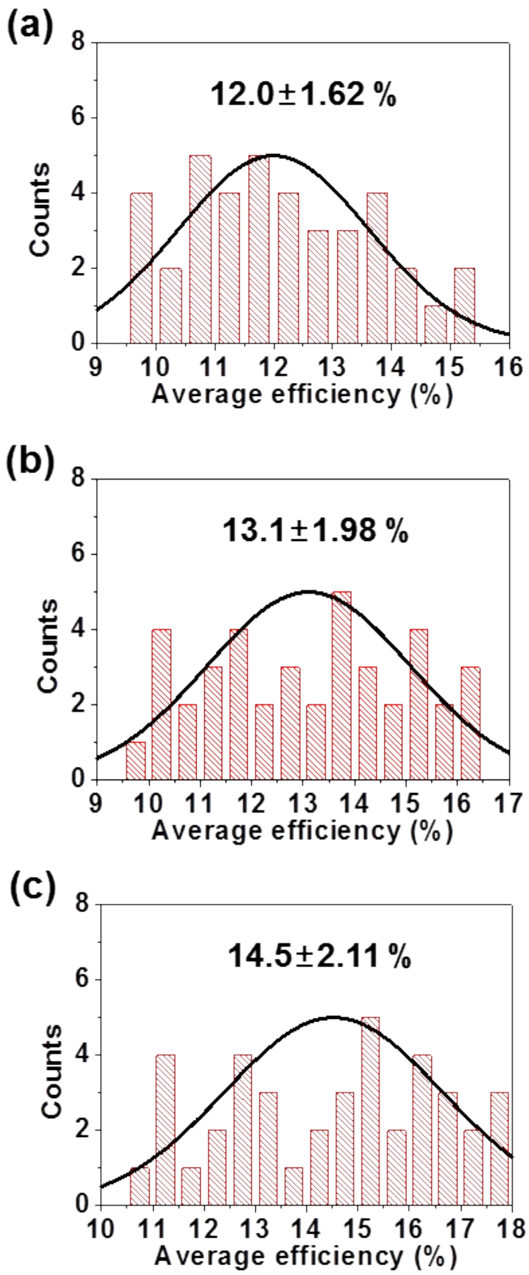

Fig. S14 Histograms of PCEs of (a) Di-TPA, (b) Tri-TPA, and (c) Tetra-TPA.

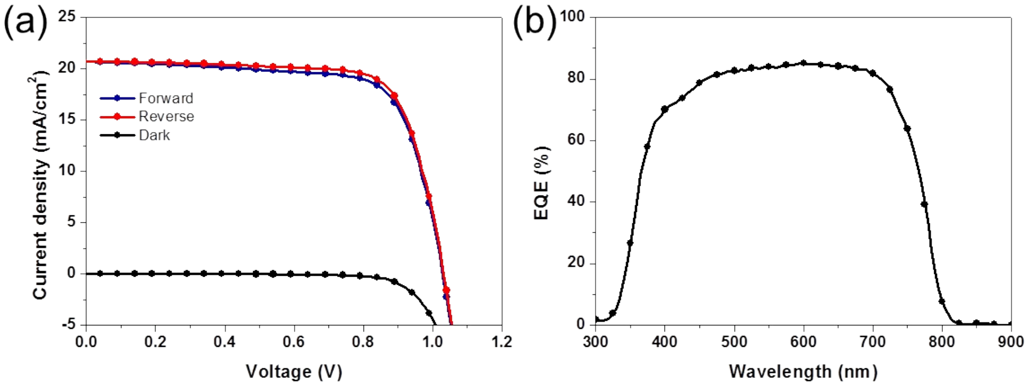

Device type	Scan direction	V_{oc}(V)	J_{sc}(mA/cm^2)	F.F(%)	η(%)
Spiro-MeOTAD	Forward	1.02	20.7	73.1	15.4
	Reverse	1.03	20.7	74.6	15.9

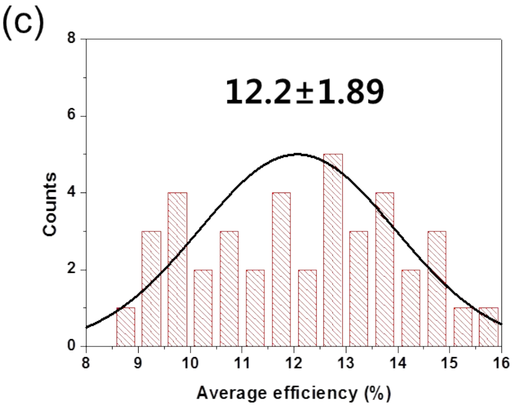

Fig. S15 (a) Photovoltaic properties of planar $MAPbI_3$ hybrid solar cells with Spiro-OMeTAD and (b) the corresponding EQE spectrum. (c) PCE histogram of 40 solar cell devices.

Reference

1 L. Chen, B. Zhang, Y. Cheng, Z. Xie, L. Wang, X. Jing and F. Wang, *Adv. Funct. Mater.,* 2010, **20**, 3143–3153.

2 G. R. Fulmer, A. J. M. Miller, N. H. Sherden, H. E. Gottlieb, A. Nudelman, B. M. Stoltz, J. E. Bercaw and K. I. Goldberg, *Organometallics,* 2010, **29**, 2176-2179.

3 G. G. Malliaras, J. R. Salem, P. J. Brock and C. Scott, *Phys. Rev. B,* 1998, **58**, 13411.

4 C. Goh, R. J. Kline, M. D. McGehee, E. N. Kadnikova and J. M. J. Frechet, *Appl. Phys. Lett.,* 2005, **86**, 122110.
